# The association between body mass index and white matter hyperintensities: a cross-sectional analysis in a Tibetan population

**DOI:** 10.3389/fneur.2026.1805682

**Published:** 2026-10-22

**Authors:** Shaoli Yao, Shi-min Bao, Yi-yin Xing, Zhen-rong Qi, Hui-ru Feng, Yuling Tan, Qi Zhang, Wei-hong Ge, Xiaorong Chen

**Affiliations:** Department of Neurology, Hospital of Chengdu Office of the People's Government of Xizang Autonomous Region, Chengdu, Sichuan, China

**Keywords:** body mass index, cerebral small vessel disease, high-altitude Tibetan population, threshold-specific binary logistic regression, white matter hyperintensities

## Abstract

**Background:**

White matter hyperintensities (WMH), an imaging hallmark of cerebral small vessel disease (CSVD), show inconsistent associations with body mass index (BMI) that vary with ethnicity, sex and metabolic factors. We examined the relationship of BMI with WMH severity (Fazekas score) and with the spatial distribution of WMH in a high-altitude Tibetan population.

**Methods:**

This cross-sectional study consecutively enrolled 499 participants hospitalized for cerebrovascular or neurological disorders between 2022 and 2025. Threshold-specific binary logistic regression, adjusted for confounders, was used to evaluate BMI–WMH associations across clinically defined severity thresholds.

**Results:**

A burden-dependent inverse association between BMI and WMH severity was observed. For total WMH burden (Fazekas score), the inverse association was more pronounced at the higher threshold (0–2 vs. 3–6: OR = 0.939, 95% CI 0.882–0.999, *p* = 0.047) than at the lower threshold (0–1 vs. 2–6: OR = 0.968, 95% CI 0.910–1.030, *p* = 0.306). For PVWMH, the association varied across thresholds (0 vs. 1–3: OR = 0.984, 95% CI 0.920–1.052, *p* = 0.639; 0–1 vs. 2–3: OR = 0.942, 95% CI 0.884–1.003, *p* = 0.064; 0–2 vs. 3: OR = 0.864, 95% CI 0.787–0.949, *p* = 0.002). DSWMH showed a similar pattern (0 vs. 1–3: OR = 1.014, 95% CI 0.945–1.088, *p* = 0.706; 0–1 vs. 2–3: OR = 0.928, 95% CI 0.872–0.988, *p* = 0.019; 0–2 vs. 3: OR = 0.874, 95% CI 0.796–0.959, *p* = 0.005). For the number of involved anatomical regions, the OR estimates were consistently below 1.0, with the strongest estimate at the highest threshold (0–3 vs. 4–10: OR = 0.947, 95% CI 0.894–1.004, *p* = 0.067).

**Conclusion:**

A burden-dependent inverse association was observed between BMI and severe WMH, strongest at the most severe WMH thresholds and particularly for periventricular WMH. These findings highlight the complex relationship between adiposity and cerebrovascular injury in high-altitude populations and underscore the need for longitudinal studies to clarify the temporal dynamics of this association.

## 1 Introduction

White matter hyperintensities (WMH), recognized neuroimaging markers of cerebral small vessel disease (CSVD), arise from complex pathological processes that include blood–brain barrier disruption (1), hypoperfusion (2), impaired amyloid clearance and axonal damage (3). WMH burden correlates with modifiable vascular factors such as hypertension (4), diabetes (5) and tobacco use (6).

The rising global prevalence of obesity—particularly in China, where more than one-third of screened adults are overweight and approximately 14% are obese (7)—has increased the relevance of body mass index (BMI) to cerebrovascular research. BMI–WMH associations vary across populations (for example, those of Australian (8), European (9–11) and North American (12) ancestry). Recent evidence highlights body-size variability as a potential driver of cerebrovascular injury (13): the cohort study by Sun et al. (13) showed that long-term ‘body-size variability’—distinct from static BMI—is independently associated with WMH progression and other CSVD neuroimaging markers, indicating that dynamic adiposity patterns may contribute to cerebrovascular injury. A large-scale analysis of 34,653 UK Biobank participants further demonstrated positive cross-sectional associations between several adiposity indices and WMH volume (14). By contrast, Driscoll et al. (15) found that obesity was associated with lower white matter lesion loads in the total, parietal and occipital regions. Shu et al. (16) reported significantly greater WMH severity in Tibetans residing at high altitude (Lhasa, Tibet; ~3,650 m) than in a Han population at low altitude (Beijing, China; ~45 m). Tibetans possess distinct physiological adaptations to chronic hypoxia, have an overweight prevalence exceeding national averages (44.2% vs. 34.8%) (7, 17), and maintain unique dietary patterns (high-fat, low-vitamin) (18)—factors that may modulate cerebrovascular risk. Nevertheless, the relationship between BMI and WMH in high-altitude Tibetan populations remains largely unexplored, representing an important gap in current understanding of CSVD.

To address this gap, we conducted the first investigation of BMI–WMH associations specifically in a high-altitude Tibetan population, with the aim of advancing understanding of CSVD pathogenesis in populations living in unique environmental settings.

## 2 Methods

### 2.1 Study population

This cross-sectional study was conducted in the Department of Neurology at the Hospital of Chengdu Office of the People’s Government of Xizang Autonomous Region between November 2022 and February 2025. Eligible participants were recruited according to clearly defined inclusion and exclusion criteria and consisted mainly of inpatients with neurological conditions, including cerebrovascular disorders, parkinsonian syndromes, vertigo, chronic headache and mood disorders such as anxiety and depression. The study protocol was approved by the hospital ethics committee (Approval No. 2022–65), and written informed consent was obtained from all participants. All procedures complied with the Declaration of Helsinki and with the Strengthening the Reporting of Observational Studies in Epidemiology (STROBE) reporting guidelines, ensuring ethical integrity and methodological quality (19).

To be eligible, participants had to: (1) be at least 35 years old; (2) be native, long-term residents of high-altitude regions of Tibet (all participants were ethnic Tibetans who reported lifelong residence at altitudes ≥ 3,000 m, defined as birth and continuous residence in the Xizang Autonomous Region; those who reported living at low altitude (< 2,500 m) for more than 12 consecutive months were excluded to distinguish lifetime adaptive traits from short-term acclimatization, and the mean residential altitude of the included cohort was 3,746.2 ± 481.6 m); and (3) have undergone standard cranial magnetic resonance imaging (MRI), including T1-weighted, T2-weighted and T2-fluid-attenuated inversion recovery (FLAIR) sequences. Participants were excluded if they had: (1) large cortical lesions, defined as cerebral infarction or hemorrhage involving at least one-third of the middle cerebral artery (MCA) territory with cortical involvement; (2) a history of intracranial infection; (3) demyelinating disease affecting the central nervous system or other white matter structures; or (4) a history of severe liver disease (Child-Pugh score ≥ 7 with clinical ascites), advanced renal impairment (chronic kidney disease stage 4–5 with estimated glomerular filtration rate [eGFR] < 30 mL/min/1.73 m^2^) or cancer, because these conditions can cause systemic metabolic disturbances that may affect the development of CSVD (20) or complicate the assessment of its biomarkers (21, 22).

After comprehensive screening, a total of 499 participants with complete brain imaging data, BMI assessments, and relevant clinical variables were ultimately included in the final analysis, as shown in Figure 1.

**Figure 1:**
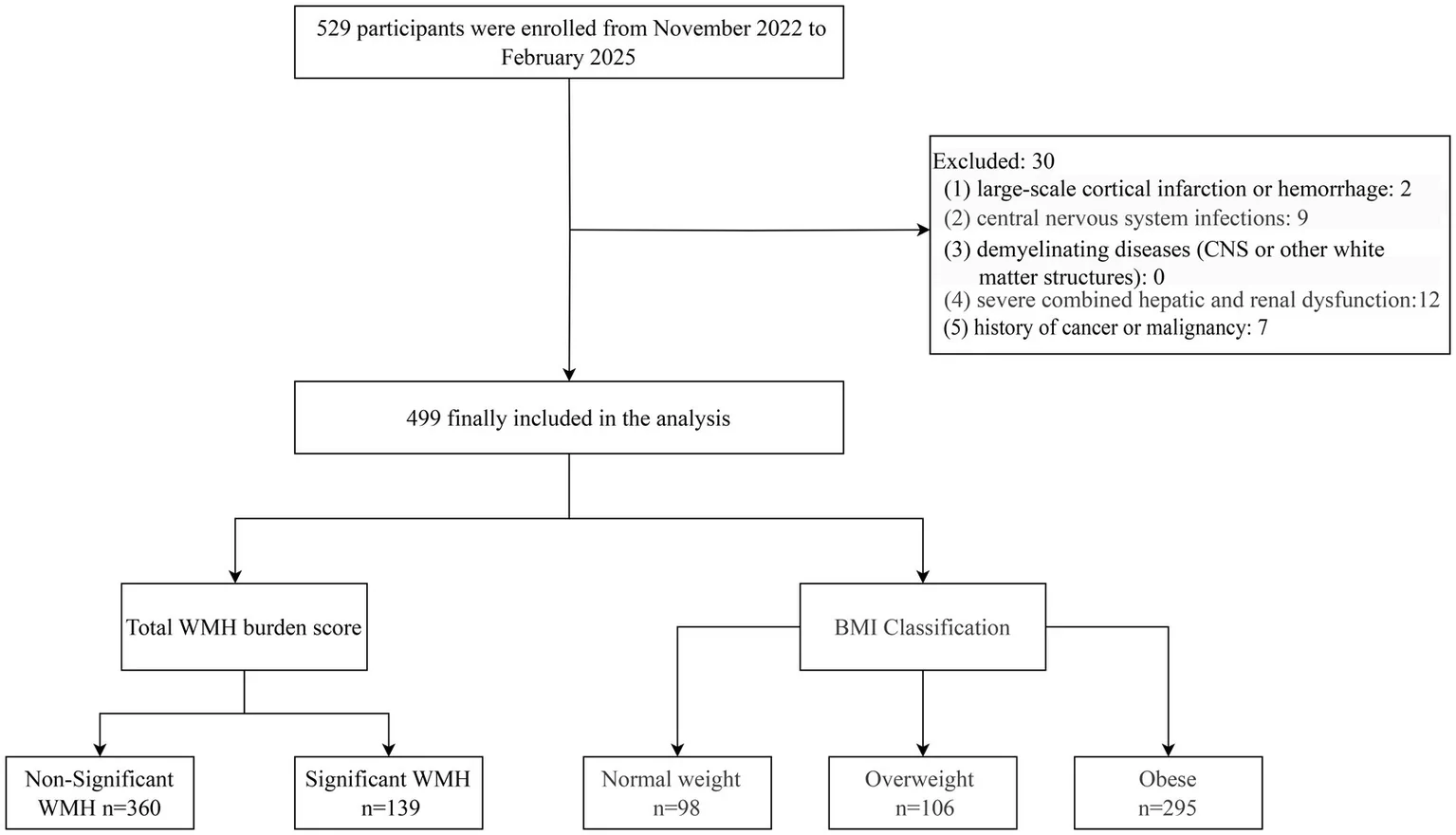
Flow diagram of the study population. BMI, body mass index; WMH, white matter hyperintensities.

### 2.2 Data collection

#### 2.2.1 Assessment of BMI

Upon admission, participants underwent standardized anthropometric assessment. Body weight and height were measured strictly according to World Health Organization (WHO) guidelines, with participants barefoot and in light clothing to minimize external influences on accuracy. BMI was calculated as weight in kilograms divided by the square of height in meters.

Given the distinct physiological characteristics of Asian populations, we used the WHO Western Pacific Region modified BMI classification for baseline descriptive characterization. Participants were categorized into three groups: normal weight (BMI < 23.0 kg/m^2^), overweight (BMI 23.0–24.9 kg/m^2^) and obesity (BMI ≥ 25.0 kg/m^2^) (23). This classification reflects population-specific features in East Asia, such as a higher body-fat percentage at lower BMI values and a greater predisposition to cardiometabolic conditions.

#### 2.2.2 Assessment of WMH

MRI data were acquired on a 3.0 T Philips MRI system (Achieva, the Netherlands) using standardized protocols that included T1-weighted, T2-weighted and T2-FLAIR sequences. WMH was evaluated according to the Standards for Reporting Vascular Changes on Neuroimaging (STRIVE) criteria (24), and severity was graded on the semi-quantitative Fazekas scale using T2-weighted or FLAIR images (25).

The Fazekas system evaluates WMH in two regions: (1) periventricular WMH (PVWMH)—grade 0, no lesions; grade 1, thin periventricular ‘caps’ or pencil-thin lining; grade 2, smooth halo-like lesions; grade 3, irregular lesions extending into the deep white matter; and (2) deep subcortical WMH (DSWMH)—grade 0, no lesions; grade 1, punctate foci; grade 2, partially confluent lesions; grade 3, large confluent lesions. Consistent with previous research, significant WMH burden was defined as PVWMH grade 3 or DSWMH grade ≥ 2 (26, 27). Total WMH burden was calculated as the sum of the PVWMH and DSWMH scores (range 0–6).

The spatial distribution of WMH was additionally assessed using a binary classification across 10 brain regions (Supplementary Figure S1): frontal, parietal, temporal and occipital lobes; periventricular region; centrum semiovale; corona radiata; internal and external capsules; and brainstem. Each region was scored as 0 (no lesion) or 1 (lesion present), and the total score represented the extent of regional WMH involvement.

All WMH ratings were performed independently by two neurologists (SY and XC) blinded to participants’ clinical data. Inter-rater reliability, assessed with weighted Cohen’s *κ* across all 499 participants, was 0.82 (95% CI 0.78–0.86) for PVWMH grading and 0.79 (95% CI 0.74–0.83) for DSWMH grading, indicating substantial agreement; disagreements (*n* = 38, 7.6%) were resolved by joint review. Intra-rater reliability, assessed by re-rating a random subset of 50 scans after a 4-week interval, yielded weighted *κ* values of 0.88 (95% CI 0.82–0.94) for rater 1 and 0.85 (95% CI 0.78–0.92) for rater 2.

#### 2.2.3 Assessment of covariates

Sociodemographic variables included sex, age, smoking status (never, former or current smoker), alcohol consumption (never, former or current drinker) and residential altitude. Clinical data on cardiovascular risk factors—hypertension, diabetes, coronary artery disease (CAD), stroke history and hyperlipidemia—were retrieved from electronic medical records. Prescribed medications were also reviewed systematically, including antihypertensive drugs, antidiabetic medications, lipid-lowering agents and antiplatelet therapy.

After an overnight fast of 8–12 h, participants underwent laboratory assessment, including fasting plasma glucose; lipid parameters (total cholesterol, triglycerides, low-density lipoprotein cholesterol [LDL-C] and high-density lipoprotein cholesterol [HDL-C]); and hepatic enzymes such as aspartate aminotransferase (AST) and alanine aminotransferase (ALT). Renal function was evaluated using the eGFR. Complete blood counts were also performed to determine red blood cell indices (e.g., hemoglobin [HGB] and hematocrit), white blood cell and platelet counts.

### 2.3 Statistical analyses

Descriptive analyses were used to summarize the demographic and clinical characteristics of the participants. Continuous variables were tested for normality with the Shapiro–Wilk test and subgrouped by BMI category and WMH burden. Normally distributed variables were presented as mean ± standard deviation and compared using independent-samples *t*-tests or one-way analysis of variance (ANOVA), as appropriate; non-normally distributed variables were presented as median and interquartile range and compared using the Mann–Whitney *U* test. Categorical variables were expressed as counts and percentages and compared using the chi-squared test or Fisher’s exact test, depending on sample size.

To investigate the associations between BMI and WMH, we used a dual analytical approach. First, multivariable logistic regression was used to assess spatial-distribution patterns by modeling region-specific WMH presence (binary outcomes). Results are presented as adjusted odds ratios (ORs) with 95% confidence intervals (CIs); Model 1 was unadjusted. Covariate selection followed three principles: (a) univariate association with WMH severity at *p* < 0.05 (28); (b) established clinical risk factors for CSVD (hypertension, diabetes, age, smoking history and sex) as defined by consensus guidelines; and (c) adherence to the empirical 10:1 events-per-variable standard to ensure model stability (29) and an interpretable number of features. Accordingly, the 10 most clinically and statistically relevant covariates were selected for Model 2: age, sex, smoking status, alcohol consumption, CAD history, stroke history, hypertension, diabetes, antihypertensive medication use and hemoglobin level.

Second, because WMH burden and the number of involved regions were non-normally distributed (Shapiro–Wilk *p* < 0.001; Supplementary Figure S2; Supplementary Table S1), we used threshold-specific binary logistic regression to analyze the ordinal WMH outcomes (Fazekas score, periventricular WMH grade, deep white matter WMH grade and number of involved anatomical regions). This approach avoids the proportional-odds assumption by allowing the effect of BMI to vary freely across severity thresholds. For each ordinal outcome, sequential binary logistic models were fitted at clinically meaningful cut-offs (26, 27): Fazekas score (0–1 vs. 2–6; 0–2 vs. 3–6), PVWMH (0 vs. 1–3; 0–1 vs. 2–3; 0–2 vs. 3), DSWMH (0 vs. 1–3; 0–1 vs. 2–3; 0–2 vs. 3) and anatomical region count (0–1 vs. 2–10; 0–2 vs. 3–10; 0–3 vs. 4–10). These threshold-specific analyses were pre-specified as complementary tests of the burden-dependent pattern of the BMI–WMH association; interpretation therefore emphasizes the consistency of the OR trend across thresholds rather than threshold-specific *p*-values. All binary logistic models were adjusted for age, sex, smoking status, alcohol consumption, CAD history, stroke, hypertension, diabetes, antihypertensive medication use and hemoglobin level. Results are presented as adjusted ORs with 95% CIs, where OR < 1 indicates an inverse association (higher BMI associated with lower odds of more severe WMH).

This study is an exploratory analysis derived from our parent project on risk factors for CSVD, which was designed primarily to assess CSVD prevalence in a population aged ≥ 35 years using standard prevalence-study methodology. The target sample size (*n* = 400) was calibrated to provide 80% power to detect a CSVD prevalence of 3.5% (α = 0.05, adjusted for attrition); 499 participants were ultimately enrolled. Exploratory analyses met the events-per-variable criterion (minimum 10:1) (29), ensuring model stability.

To explore potential mediating pathways, we conducted an exploratory product-of-coefficients mediation analysis of three candidate mediators—triglycerides, HDL cholesterol and hypertension—selected for their established roles in the obesity–cerebrovascular disease pathway. For each mediator, we estimated the indirect effect of BMI on severe WMH (Fazekas 0–2 vs. 3–6), with statistical inference based on the Sobel test and bias-corrected bootstrap 95% CIs (5,000 resamples). All mediation models were adjusted for the full covariate set (age, sex, smoking, alcohol, CAD, stroke, hypertension, diabetes, antihypertensive therapy and HGB). We emphasize that these analyses are purely exploratory and cross-sectional; causal interpretation is precluded because the exposure, mediators and outcome were measured concurrently.

To test formally whether hypoxia-related factors modified the inverse BMI–WMH association in deep brain regions, we performed interaction analyses. First, a BMI × altitude interaction term was added to the multivariable logistic models for corona radiata and brainstem WMH. Second, given the elevated hemoglobin levels typical of this high-altitude population, we performed HGB-stratified analyses using a median split (147 g/L) with a parsimonious covariate set (age, sex, hypertension) to avoid model non-convergence due to the low event counts for these regional outcomes.

All statistical analyses were performed with R version 4.3.1 (^1^The R Foundation) and Free Statistics software version 2.5.1 (30). Two-tailed tests were used, and *p* < 0.05 was considered statistically significant.

## 3 Results

### 3.1 Characteristics of the participants

A total of 499 participants were included. Significant WMH burden was identified in 27.9% (139/499), and the overall prevalence of obesity was 59.1% (295/499); using the WHO diagnostic criterion for obesity (BMI ≥ 28 kg/m^2^) (31), the prevalence was 29.5% (147/499). The highest regional WMH frequencies were in the frontal lobe (82.6%) and the periventricular region (81.2%); other affected regions included the parietal lobe (53.5%) and the centrum semiovale (24.6%). Comprehensive regional data are provided in Supplementary Table S2.

Table 1 summarizes the characteristics of the 499 participants stratified by BMI category. Mean age was 57.7 years (SD 11.7) and 55.7% (278/499) were male. Obese participants had significantly higher hemoglobin concentrations than the other BMI groups (*p* = 0.023). WMH prevalence differed significantly across BMI categories in several regions—the occipital lobe (*p* = 0.004), corona radiata (*p* < 0.001), internal capsule (*p* < 0.001) and brainstem (*p* = 0.002)—and the number of affected anatomical regions also varied significantly (*p* = 0.049).Triglycerides (*p* < 0.001), HDL-C (*p* = 0.005), LDL-C (*p* = 0.002), total cholesterol (*p* = 0.014) and fasting plasma glucose (*p* = 0.011) also differed significantly across BMI categories, whereas the remaining characteristics did not differ significantly. The characteristics of the 499 participants stratified by WMH burden are provided in Supplementary Table S3: participants with severe WMH were significantly older (65.3 ± 9.7 vs. 54.7 ± 11 years; *p* < 0.001), had a higher prevalence of stroke (18.7% vs. 6.9%; *p* < 0.001) and hypertension (71.9% vs. 41.4%; *p* < 0.001), and had a lower BMI (25.4 ± 3.7 vs. 26.4 ± 4 kg/m^2^, *p* = 0.012).

**Table 1:** Characteristics of the 499 study participants.

Variables	Total (*n* = 499)	Normal weight (*n* = 98)	People with overweight (*n* = 106)	People with obesity (*n* = 295)	*p*
Age, year	57.7 ± 11.7	57.9 ± 12.2	58.6 ± 11.1	57.2 ± 11.7	0.584
Altitude, meters	3746.2 ± 481.6	3703.3 ± 406.8	3727.7 ± 433.1	3767.1 ± 519.7	0.476
WMH-involved regions	3.0 (2.0, 4.0)	3.0 (2.0, 4.0)	3.0 (2.0, 4.0)	3.0 (2.0, 3.5)	0.049
HGB, g/L	148.1 ± 24.2	144.5 ± 23.1	144.4 ± 25.1	150.5 ± 24.0	0.023
WBC, 10^9^/L	5.8 ± 1.7	5.6 ± 1.8	6.0 ± 2.0	5.7 ± 1.6	0.309
FPG, mmol/L	4.9 (4.5, 5.6)	4.8 (4.4, 5.2)	4.9 (4.5, 5.7)	5.0 (4.6, 5.6)	0.011
Triglycerides, mmol/L	1.3 ± 0.7	1.1 ± 0.4	1.3 ± 0.7	1.4 ± 0.7	< 0.001
HDL-C, mmol/L	1.1 ± 0.3	1.2 ± 0.3	1.2 ± 0.3	1.1 ± 0.3	0.005
LDL-C, mmol/L	2.5 ± 0.8	2.3 ± 0.7	2.7 ± 0.9	2.6 ± 0.8	0.002
Total cholesterol, mmol/L	4.2 ± 1.0	4.0 ± 0.9	4.4 ± 1.1	4.2 ± 1.0	0.014
Sex, *n* (%)					0.508
Female	221 (44.3)	48 (49)	48 (45.3)	125 (42.4)	
Male	278 (55.7)	50 (51)	58 (54.7)	170 (57.6)	
Living alone, *n* (%)					0.752
No	436 (87.4)	87 (88.8)	94 (88.7)	255 (86.4)	
Yes	63 (12.6)	11 (11.2)	12 (11.3)	40 (13.6)	
Smoking status, *n* (%)					0.747
Never	307 (61.5)	59 (60.2)	63 (59.4)	185 (62.7)	
Former	105 (21.0)	18 (18.4)	24 (22.6)	63 (21.4)	
Current smokers	87 (17.4)	21 (21.4)	19 (17.9)	47 (15.9)	
Alcohol status, *n* (%)					0.682
Never	283 (56.7)	58 (59.2)	56 (52.8)	169 (57.3)	
Former	127 (25.5)	25 (25.5)	32 (30.2)	70 (23.7)	
Current drinkers	89 (17.8)	15 (15.3)	18 (17)	56 (19)	
Hypertension, *n* (%)					0.432
No	250 (50.1)	51 (52)	58 (54.7)	141 (47.8)	
Yes	249 (49.9)	47 (48)	48 (45.3)	154 (52.2)	
Diabetes, *n* (%)					0.683
No	431 (86.4)	82 (83.7)	92 (86.8)	257 (87.1)	
Yes	68 (13.6)	16 (16.3)	14 (13.2)	38 (12.9)	
CAD, *n* (%)					0.569
No	475 (95.2)	95 (96.9)	102 (96.2)	278 (94.2)	
Yes	24 (4.8)	3 (3.1)	4 (3.8)	17 (5.8)	
Hyperlipidemia, *n* (%)					0.227
No	464 (93.0)	95 (96.9)	98 (92.5)	271 (91.9)	
Yes	35 (7.0)	3 (3.1)	8 (7.5)	24 (8.1)	
Stroke, *n* (%)					0.413
No	448 (89.8)	85 (86.7)	94 (88.7)	269 (91.2)	
Yes	51 (10.2)	13 (13.3)	12 (11.3)	26 (8.8)	
WMH anatomical distribution Significant WMH burden, *n* (%)					0.101
No	360 (72.1)	64 (65.3)	73 (68.9)	223 (75.6)	
Yes	139 (27.9)	34 (34.7)	33 (31.1)	72 (24.4)	
Periventricular, *n* (%)					0.095
No	94 (18.8)	17 (17.3)	13 (12.3)	64 (21.7)	
Yes	405 (81.2)	81 (82.7)	93 (87.7)	231 (78.3)	
Frontal lobe, *n* (%)					0.574
No	87 (17.4)	19 (19.4)	15 (14.2)	53 (18)	
Yes	412 (82.6)	79 (80.6)	91 (85.8)	242 (82)	
Parietal lobe, *n* (%)					0.064
No	232 (46.5)	39 (39.8)	43 (40.6)	150 (50.8)	
Yes	267 (53.5)	59 (60.2)	63 (59.4)	145 (49.2)	
Temporal lobe, *n* (%)					0.240
No	436 (87.4)	81 (82.7)	92 (86.8)	263 (89.2)	
Yes	63 (12.6)	17 (17.3)	14 (13.2)	32 (10.8)	
Occipital lobe, *n* (%)					0.004
No	422 (84.6)	73 (74.5)	96 (90.6)	253 (85.8)	
Yes	77 (15.4)	25 (25.5)	10 (9.4)	42 (14.2)	
Centrum semiovale, *n* (%)					0.058
No	376 (75.4)	65 (66.3)	80 (75.5)	231 (78.3)	
Yes	123 (24.6)	33 (33.7)	26 (24.5)	64 (21.7)	
Corona radiata, *n* (%)					< 0.001
No	475 (95.2)	85 (86.7)	102 (96.2)	288 (97.6)	
Yes	24 (4.8)	13 (13.3)	4 (3.8)	7 (2.4)	
Internal capsule, *n* (%)					< 0.001
No	479 (96.0)	88 (89.8)	106 (100)	285 (96.6)	
Yes	20 (4.0)	10 (10.2)	0 (0)	10 (3.4)	
External capsule, *n* (%)					0.213
No	461 (92.4)	87 (88.8)	101 (95.3)	273 (92.5)	
Yes	38 (7.6)	11 (11.2)	5 (4.7)	22 (7.5)	
Brain stem, *n* (%)					0.002
No	482 (96.6)	90 (91.8)	101 (95.3)	291 (98.6)	
Yes	17 (3.4)	8 (8.2)	5 (4.7)	4 (1.4)	

The data are shown as the mean ± standard deviation, median (interquartile range), or frequency (%), as appropriate.

HGB, hemoglobin; WBC, white blood cell; FPG, fasting plasma glucose; LDL-C, low-density lipoprotein cholesterol; HDL-C, high-density lipoprotein cholesterol; CAD, coronary artery disease; WMH, white matter hyperintensities; BMI, body mass index.

When applying the WHO global standard (31) (obesity = BMI ≥ 28.0 kg/m^2^), the obesity prevalence in our study population was 29.5%.

### 3.2 Association of BMI and WMH burden among 499 study participants

Threshold-specific binary logistic regression revealed a consistent inverse association between higher BMI and lower odds of more severe WMH that strengthened with increasing severity threshold (Table 2). For total WMH (Fazekas score), the OR shifted from 0.968 (95% CI 0.910–1.030) at the lower threshold (0–1 vs. 2–6) to 0.939 (95% CI 0.882–0.999) at the higher threshold (0–2 vs. 3–6). A monotonic inverse pattern across the three thresholds was observed for PVWMH (0 vs. 1–3: OR = 0.984, 95% CI 0.920–1.052; 0–1 vs. 2–3: OR = 0.942, 95% CI 0.884–1.003; 0–2 vs. 3: OR = 0.864, 95% CI 0.787–0.949) and DSWMH (0 vs. 1–3: OR = 1.014, 95% CI 0.945–1.088; 0–1 vs. 2–3: OR = 0.928, 95% CI 0.872–0.988; 0–2 vs. 3: OR = 0.874, 95% CI 0.796–0.959). The anatomical region count showed a similar inverse pattern, with the strongest estimate at the highest threshold (0–3 vs. 4–10: OR = 0.947, 95% CI 0.894–1.004). Overall, the inverse association was most pronounced at the most severe WMH thresholds, particularly for periventricular WMH.

**Table 2:** Threshold-specific binary logistic regression analysis of BMI and WMH burden.

Outcome variables	Threshold	OR	95% CI	*p*
Total WMH burden (Fazekas scores)	0–1 vs. 2–6	0.968	0.910–1.030	0.306
	0–2 vs. 3–6	0.939	0.882–0.999	0.047
PVWMH Fazekas scores	0 vs. 1–3	0.984	0.920–1.052	0.639
	0–1 vs. 2–3	0.942	0.884–1.003	0.064
	0–2 vs. 3	0.864	0.787–0.949	0.002
DSWMH Fazekas scores	0 vs. 1–3	1.014	0.945–1.088	0.706
	0–1 vs. 2–3	0.928	0.872–0.988	0.019
	0–2 vs. 3	0.874	0.796–0.959	0.005
The number of affected anatomical regions	0–1 vs. 2–10	0.965	0.906–1.028	0.268
	0–2 vs. 3–10	0.975	0.927–1.026	0.326
	0–3 vs. 4–10	0.947	0.894–1.004	0.067

The model adjusted for age, sex, smoking status, alcohol consumption, history of coronary artery disease, stroke, hypertension, diabetes, use of antihypertensive medications, and hemoglobin level. OR < 1 indicates an inverse association (higher BMI associated with lower odds of more severe WMH).

### 3.3 The association between BMI and WMH in different brain regions

Multivariable logistic regression, adjusted for age and comorbidities, explored the associations between BMI and region-specific WMH (Table 3). Significant inverse relationships were observed between BMI and WMH presence in both the corona radiata (OR = 0.76, 95% CI 0.65–0.88, *p* < 0.001) and the brainstem (OR = 0.74, 95% CI 0.62–0.87, *p* < 0.001).

**Table 3:** The association between BMI and WMH in different brain regions.

Brain regions	Model 1		Model 2	
	OR (95% CI)	*p* value	OR (95% CI)	*p* value
Periventricular	0.97 (0.92 ~ 1.03)	0.370	0.96 (0.90 ~ 1.03)	0.237
Frontal lobe	1.01 (0.95 ~ 1.07)	0.698	1.00 (0.94 ~ 1.07)	0.925
Parietal lobe	0.97 (0.93 ~ 1.01)	0.178	0.97 (0.92 ~ 1.02)	0.172
Temporal lobe	0.97 (0.93 ~ 1.01)	0.178	0.96 (0.89 ~ 1.03)	0.236
Occipital lobe	0.94 (0.88 ~ 1.01)	0.075	0.94 (0.87 ~ 1.00)	0.065
Centrum semiovale	0.97 (0.92 ~ 1.02)	0.298	0.97 (0.92 ~ 1.03)	0.376
Corona radiata	0.78 (0.69 ~ 0.88)	<0.001	0.76 (0.65 ~ 0.88)	<0.001
Internal capsule	0.90 (0.79 ~ 1.01)	0.085	0.90 (0.79 ~ 1.03)	0.141
External capsule	0.94 (0.87 ~ 1.03)	0.205	0.94 (0.85 ~ 1.03)	0.177
Brain stem	0.75 (0.65 ~ 0.87)	<0.001	0.74 (0.62 ~ 0.87)	<0.001

Model 1: Adjusted for nothing.

Model 2: Adjusted for age, sex, smoking status, alcohol status, CAD history, stroke history, hypertension history, diabetes, use of antihypertensive medications, and HGB.

Since no WMH in cerebellum were observed in our study population, the association between BMI and cerebellar WMH was not performed.

BMI, body mass index; CAD, coronary artery disease; WMH, white matter hyperintensities; OR, odds ratio; CI, confidence interval.

### 3.4 Supplementary analyses

In an exploratory, hypothesis-generating mediation analysis, reported in full in the Supplementary Table S4, triglycerides, HDL cholesterol and hypertension were examined as candidate mediators of the BMI–severe WMH association; no statistically significant indirect effects were found. The indirect effect was −0.0010 (95% CI − 0.0107 to 0.0078, *p* = 0.838) through triglycerides, 0.0011 (95% CI − 0.0069 to 0.0108, *p* = 0.762) through HDL-C and 0.0284 (95% CI − 0.0212 to 0.0779, *p* = 0.262) through hypertension. Because the exposure, mediator and outcome were measured concurrently, these estimates are exploratory only and cannot establish temporal or causal mediation.

Interaction analyses (Supplementary Table S5) revealed no significant modification of the BMI–WMH association by altitude for either the corona radiata (BMI × altitude OR = 0.9999, 95% CI 0.9995–1.0003, *p* = 0.556) or the brainstem (OR = 0.9998, 95% CI 0.9994–1.0001, *p* = 0.228). In HGB-stratified analyses, the inverse BMI–WMH association was consistent across both strata: for the corona radiata, low HGB OR = 0.74 (95% CI 0.60–0.90, *p* = 0.003) and high HGB OR = 0.78 (95% CI 0.65–0.95, *p* = 0.014); for the brainstem, low HGB OR = 0.74 (95% CI 0.60–0.92, *p* = 0.005) and high HGB OR = 0.72 (95% CI 0.56–0.93, *p* = 0.013).

## 4 Discussion

To our knowledge, this is the first systematic investigation of the association between BMI and WMH burden in a Tibetan population residing above 3,600 m (mean ± SD 3,746.2 ± 481.6 m). Using standardized Fazekas visual grading of WMH severity combined with threshold-specific binary logistic regression, we identified a burden-dependent inverse association between BMI and WMH burden.

### 4.1 Burden-dependent inverse association between BMI and WMH

The core finding of this study—a burden-dependent inverse association between higher BMI and lower odds of severe WMH—extends the evolving understanding of the obesity paradox in cerebrovascular disease. European and American cohort studies have consistently identified elevated BMI as a significant risk factor for WMH progression (9, 10) through obesity-driven chronic low-grade inflammation, endothelial dysfunction and insulin resistance (32, 33); East Asian evidence has corroborated this relationship (11); and a large-scale analysis of 34,653 UK Biobank participants further demonstrated positive cross-sectional associations between several adiposity indices and WMH volume (14). Our results, however, indicate a more complex pattern in high-altitude Tibetan populations. The inverse association was most pronounced at the highest WMH severity thresholds (PVWMH and DSWMH grade 0–2 vs. 3), consistent with a burden-dependent pattern rather than a uniform inverse association across all severity levels. Notably, this finding aligns with the earlier report by Driscoll et al. (15), who observed that obesity was associated with lower white matter lesion loads in the total, parietal and occipital regions. That observation, seldom replicated in general populations, finds a parallel in our high-altitude Tibetan cohort, thereby expanding the known boundary of the obesity paradox. In our cohort, the inverse association at the highest severity thresholds may reflect the unique high-altitude metabolic environment, in which chronic hypoxia induces adaptive physiological changes that alter the relationship between adiposity and cerebrovascular injury.

The mechanisms underlying this burden-dependent inverse association remain incompletely understood. We propose the following integrated pathway. Tibetan populations have resided at high altitude (>3,000 m) for millennia and harbor unique EPAS1/HIF-2α genetic adaptations (34, 35). Chronic hypoxia upregulates vascular endothelial growth factor (VEGF), promotes angiogenesis, and enhances cerebral collateral circulation (36–38). Higher BMI may provide the metabolic reserve necessary to sustain these protective mechanisms. Consistent with this, Korean studies have reported an inverse association between obesity and poor outcomes after ischemic stroke, particularly in the small vessel disease subtype (39). A multi-center review further observed that obesity is associated with lower mortality following intracerebral hemorrhage (40). High-altitude environments exert selective metabolic pressure, favoring a metabolically healthy obesity phenotype (41). Indeed, Tibetan populations exhibit a lower prevalence of metabolic syndrome than low-altitude Han Chinese at comparable BMI levels (42), suggesting that a “healthy obesity” phenotype in this context may not increase—and may even reduce—the risk of cerebrovascular injury. Multiple studies have demonstrated a positive association between BMI and favorable stroke outcomes (43, 44). In harsh environments, higher BMI represents better nutritional status and physiological reserve; during acute or chronic brain injury, greater energy reserves may buffer metabolic crises (45). Furthermore, adipose tissue secretes adiponectin, which possesses anti-inflammatory and antioxidant properties (46), while leptin exerts neuroprotective effects under specific conditions (47). These EPAS1/HIF-2α variants attenuate the excessive erythropoiesis and blood hyperviscosity that develop in lowlanders exposed to chronic hypoxia, thereby helping to preserve microvascular integrity (48, 49). Chronic hypoxia may also remodel adipose tissue, promoting a brown-like phenotype and shifting the adipokine secretion profile toward one that supports, rather than impairs, cerebrovascular function (50); in this setting, a higher BMI may act as a metabolic reserve that sustains perfusion of watershed territories such as the corona radiata. These mechanisms remain hypothetical and require further validation.

Our exploratory, cross-sectional mediation analysis did not identify statistically significant indirect effects through triglycerides, HDL cholesterol or hypertension; because the exposure, mediator and outcome were measured at the same time, these estimates cannot distinguish direct from indirect effects. This analysis is therefore presented only to generate hypotheses and should not be read as evidence for or against any causal sequence. Pathways other than conventional cardiometabolic risk factors—such as hemodynamic adaptation, hypoxia-inducible factor signaling or adipokine-mediated neuroprotection—warrant investigation in longitudinal studies with repeated measures rather than being inferred from these cross-sectional data.

Obstructive sleep apnoea (OSA) is highly prevalent at high altitude, where hypobaric hypoxia and central respiratory instability coexist, and obesity is its principal anatomical driver (51, 52). OSA independently aggravates cerebral small vessel disease and white matter hyperintensity burden through intermittent nocturnal hypoxemia, oxidative stress, blood–brain barrier disruption and sympathetic overactivity (51, 53, 54). In the present study we did not assess OSA: neither polysomnography data nor questionnaire-based screening were available. Because obese participants are at the greatest risk of undiagnosed OSA (52), the inverse BMI–WMH association observed here must be interpreted with particular caution, since OSA is a plausible unmeasured confounder that could attenuate, or even reverse this association. Prospective studies incorporating objective sleep assessment are needed to clarify this pathway.

### 4.2 The association between BMI and WMH in different brain regions

Our analysis further revealed an inverse relationship between BMI and WMH burden in the corona radiata and brainstem—regions not conventionally associated with the obesity paradox. This regional specificity warrants consideration. The corona radiata, a critical white matter tract connecting cortical and subcortical structures, exhibits heightened sensitivity to hemodynamic fluctuations. While obesity typically confers cerebrovascular risk, in this high-altitude cohort, higher BMI may paradoxically support perfusion stability in select regions through adipokine-mediated pathways. Specifically, visceral adipose tissue releases adiponectin (46), which demonstrates anti-inflammatory and angiogenic properties that may enhance endothelial resilience against chronic hypoperfusion. Furthermore, chronic hypoxia may stimulate adipose production of protective antioxidants (e.g., superoxide dismutase 2) (55), potentially counteracting oxidative stress in vulnerable watershed areas. To further characterize this region-specific inverse association, we performed interaction and stratified analyses. Altitude did not significantly modify the BMI–WMH association in either the corona radiata or brainstem, indicating that finer altitude gradients within this high-altitude sample do not materially alter the relationship. Similarly, hemoglobin-stratified analyses showed a consistent inverse BMI–WMH association across both low and high hemoglobin groups in both the corona radiata and brainstem, suggesting that hemoglobin level is not a major modifier of the deep brain inverse association. However, the precise mechanisms underlying this region-specific inverse association remain incompletely characterized. We acknowledge that a key limitation in interpreting this potential protective effect is the lack of direct measurement of adipokines (e.g., adiponectin, angiopoietin-like protein 4) or biomarkers of oxidative stress/antioxidant capacity within this cohort. While the proposed adipokine-mediated pathway is biologically plausible based on extant literature (46, 55), it remains speculative and necessitates further validation. Future studies should prioritize longitudinal adipokine profiling coupled with advanced neuroimaging techniques such as arterial spin labeling to quantify region-specific cerebral blood flow and microvascular adaptations under chronic hypoxic exposure.

### 4.3 Strengths and limitations

This study has several notable strengths. First, we identified a burden-dependent inverse association between BMI and severe WMH, with the strongest effect at the most severe WMH thresholds, reporting a novel pattern that addresses an important gap in the epidemiology of high-altitude cerebral small vessel disease. Second, through meticulous WMH phenotyping in a Tibetan cohort, we provide population-specific pathophysiological insights that highlight neurovascular vulnerability under chronic hypoxia. Third, the differential BMI–WMH associations across severity strata suggest stage-specific biological susceptibility, providing a foundation for future longitudinal mechanistic investigations.

Several limitations warrant consideration. First, the hospital-based sampling of neurological inpatients at a single center is a major source of selection bias. Participants were admitted for cerebrovascular or neurological disorders, and their reasons for admission are themselves associated with both adiposity and cerebrovascular pathology. To allow readers to judge this directly, we present the primary discharge diagnoses stratified by BMI category in Supplementary Table S6. The proportion discharged with cerebrovascular disease—the category most closely linked to severe white matter disease—was similar across the normal-weight, overweight and obese groups (34.7%, 30.2 and 32.2%; *p* = 0.794), whereas dizziness was significantly less frequent in normal-weight participants (1.0% vs. 12.3 and 8.1%; *p* = 0.004). Although these findings argue against a large differential admission effect by cerebrovascular indication, we cannot exclude residual selection bias, and our results may not be generalizable to the community-dwelling Tibetan population. Validation in population-based, multi-center cohorts with representative sampling frames is essential before these results can inform clinical or public-health decisions. Second, this study used BMI as the sole adiposity measure. Although waist circumference (WC) provides superior characterization of visceral fat distribution—a key determinant of metabolic risk (41)—our retrospective database did not include WC data, precluding validation using the lipid accumulation product or visceral adiposity index. Nonetheless, BMI and WC are strongly correlated in general populations (r ≈ 0.70–0.80) (56), and BMI has been widely validated as a surrogate for visceral adiposity in Asian populations, who exhibit higher body fat at lower BMI levels compared with European populations (57, 58). Future work should incorporate anthropometric measurements to better delineate the relationship between adipose tissue distribution and WMH. Third, as a cross-sectional investigation, this study cannot establish temporal sequence or causality between BMI and WMH. This limitation applies equally to our exploratory mediation analyses, because exposure, mediators, and outcome were measured concurrently. Cross-sectional mediation data cannot disentangle direct from indirect causal effects, and estimates of indirect pathways derived from such data may be biased (59, 60). We therefore caution against causal interpretation of these mediation results. Longitudinal studies incorporating repeated measurements of BMI, metabolic biomarkers, and WMH progression are needed to properly characterize temporal mediation pathways in order to explore the metabolic mechanisms underlying the inverse association between BMI and WMH. Fourth, despite comprehensive adjustment for clinical and demographic covariates, our analysis lacked data on some important confounders, such as sleep-disordered breathing. OSA is highly prevalent at high altitude and in obesity and independently promotes white matter hyperintensities (51, 53, 54), but we could not adjust for it because such data were not available. The absence of OSA assessment is an important unmeasured confounder that could bias the observed inverse association; therefore, in future studies, we will prioritize OSA screening. Other unmeasured factors, including physical activity and diet, may also contribute residual confounding, so the associations reported here warrant cautious interpretation. Finally, while the Fazekas scale offers established clinical utility and shows strong concordance with quantitative measurements (correlation coefficients ranging from 0.74 to 0.91 across raters) (61), volumetric quantification would enhance sensitivity for detecting subtle WMH changes (62, 63). The visual scale is nonetheless subject to ceiling effects and bears a non-linear relationship to true continuous lesion volume, particularly at the highest severity grades (64, 65), such that a Fazekas score of 3 encompasses a wide range of absolute confluent lesion volumes; the burden-dependent inverse association observed at this grade may therefore be influenced by the scale’s inability to discriminate among markedly different lesion volumes. The absence of volumetric WMH analysis in the present study is attributable to technical constraints, including the lack of an automated segmentation pipeline validated for our FLAIR acquisition parameters and the absence of three-dimensional T1-weighted sequences required for reliable intracranial volume normalization. Automated volumetric segmentation on high-resolution T1-weighted and FLAIR images, together with diffusion-tensor assessment of the normal-appearing white matter, would provide a more sensitive and continuous measure of lesion burden and microstructural integrity.

## 5 Conclusion

This cross-sectional study reveals a burden-dependent inverse association between BMI and severe WMH among high-altitude Tibetan residents, contributing to the evolving understanding of the obesity paradox in cerebrovascular disease. The inverse association was most pronounced at the highest WMH severity thresholds, particularly for periventricular WMH, suggesting that the BMI–WMH relationship may vary with disease stage. These findings should be interpreted in the context of the cross-sectional design, which precludes causal inference. Future prospective studies incorporating longitudinal BMI and WMH measurements, volumetric quantification, and metabolomic profiling are warranted to elucidate the mechanisms underlying this inverse association in high-altitude populations. Finally, these findings are limited by the hospital-based cross-sectional design, by potential selection bias, and by the absence of data on obstructive sleep apnoea, an important unmeasured confounder.

## Data Availability

The datasets generated or analyzed during the current study are not publicly available due to ethical restrictions but are available from the corresponding author upon reasonable request. Requests to access the datasets should be directed to 15208354834@163.com.

## Footnotes

1
http://www.R-project.org
