# Supplementary material for: The association between body mass index and white matter hyperintensities: a cross-sectional analysis in a Tibetan population
Source: Front Neurol. 2026 Oct 22;17:1805682. doi: 10.3389/fneur.2026.1805682 (PMC13761218; doi:10.3389/fneur.2026.1805682)
Supplement: Supplementary file 1 [file Data_Sheet_1.PDF]

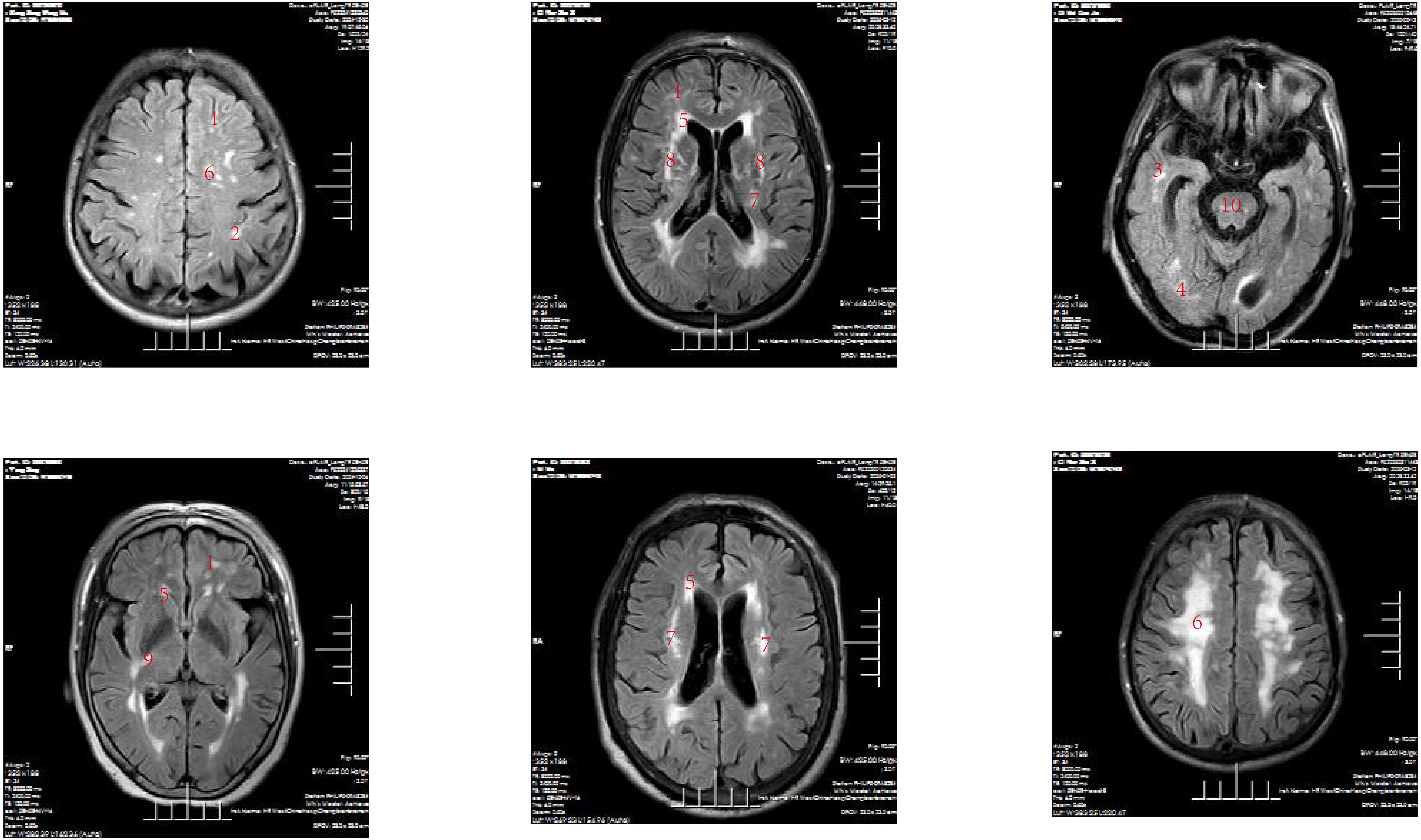
1
6
2
1
5
8
8
7
3
10
4
1
5
9
5
7
7
6
