# Supplementary material for: The association between body mass index and white matter hyperintensities: a cross-sectional analysis in a Tibetan population
Source: Front Neurol. 2026 Oct 22;17:1805682. doi: 10.3389/fneur.2026.1805682 (PMC13761218; doi:10.3389/fneur.2026.1805682)
Supplement: Supplementary file 2 [file Data_Sheet_2.PDF]

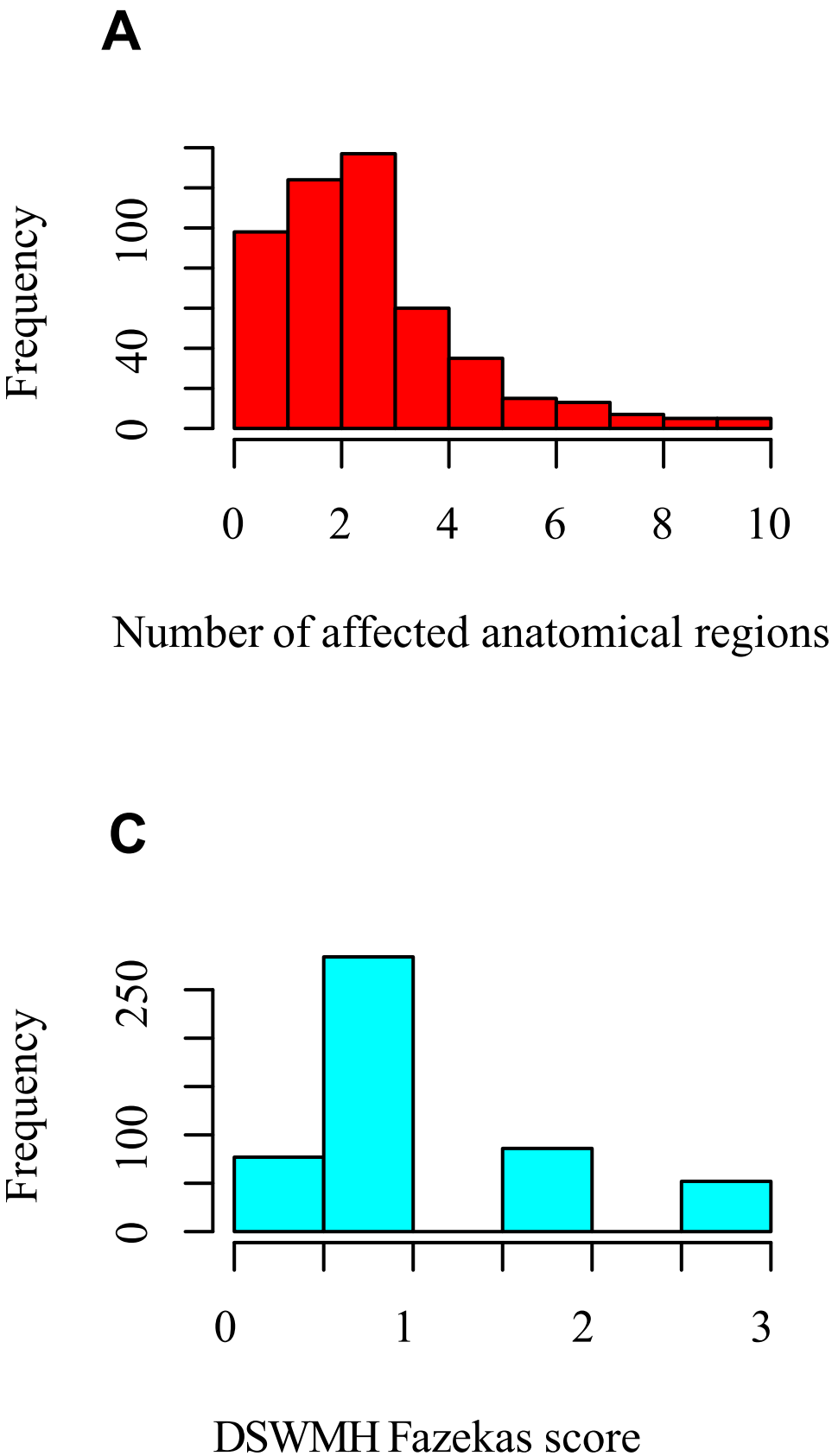
A
Frequency
0
40
100
0
2
4
6
8
10
Number of affected anatomical regions
B
Frequency
0
100
250
0
1
2
3
4
5
6
Total Fazekas score
C
Frequency
0
100
250
0
1
2
3
DSWMH Fazekas score
D
Frequency
0
100
250
0
1
2
3
PVWMH Fazekas score
