# Supplementary material for: The association between body mass index and white matter hyperintensities: a cross-sectional analysis in a Tibetan population
Source: Front Neurol. 2026 Oct 22;17:1805682. doi: 10.3389/fneur.2026.1805682 (PMC13761218; doi:10.3389/fneur.2026.1805682)
Supplement: Supplementary file 3 [file Data_Sheet_3.PDF]

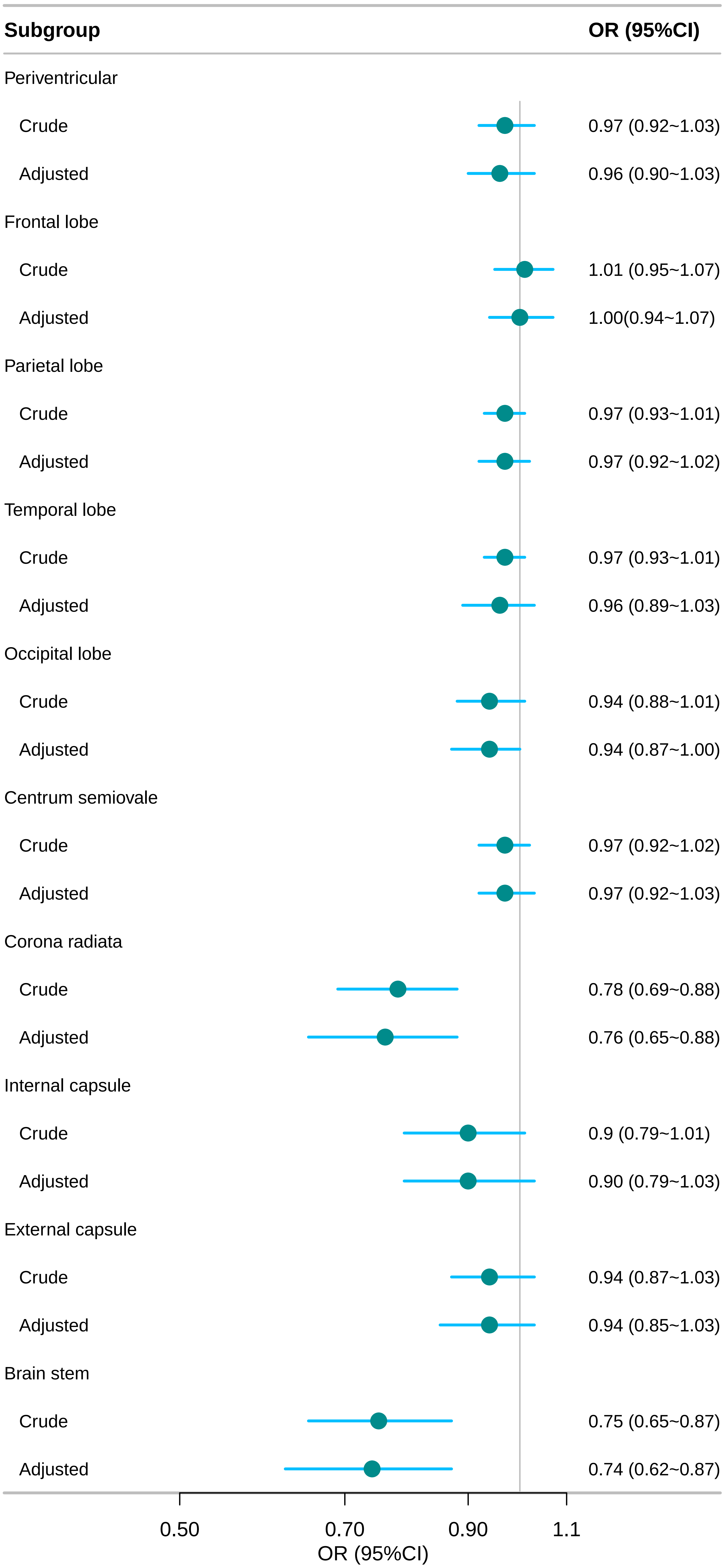

Subgroup
OR (95%CI)
Periventricular
Crude
0.97 (0.92~1.03)
Adjusted
0.96 (0.90~1.03)
Frontal lobe
Crude
1.01 (0.95~1.07)
Adjusted
1.00(0.94~1.07)
Parietal lobe
Crude
0.97 (0.93~1.01)
Adjusted
0.97 (0.92~1.02)
Temporal lobe
Crude
0.97 (0.93~1.01)
Adjusted
0.96 (0.89~1.03)
Occipital lobe
Crude
0.94 (0.88~1.01)
Adjusted
0.94 (0.87~1.00)
Centrum semiovale
Crude
0.97 (0.92~1.02)
Adjusted
0.97 (0.92~1.03)
Corona radiata
Crude
0.78 (0.69~0.88)
Adjusted
0.76 (0.65~0.88)
Internal capsule
Crude
0.9 (0.79~1.01)
Adjusted
0.90 (0.79~1.03)
External capsule
Crude
0.94 (0.87~1.03)
Adjusted
0.94 (0.85~1.03)
Brain stem
Crude
0.75 (0.65~0.87)
Adjusted
0.74 (0.62~0.87)
0.50
0.70
0.90
1.1
OR (95%CI)
