# Supplementary material for: The association between body mass index and white matter hyperintensities: a cross-sectional analysis in a Tibetan population
Source: Front Neurol. 2026 Oct 22;17:1805682. doi: 10.3389/fneur.2026.1805682 (PMC13761218; doi:10.3389/fneur.2026.1805682)
Supplement: Supplementary file 4 [file Table_1.DOCX]

| Table S1 Shapiro-Wilk Test Results for Normality Assessment of the number of affected anatomical regions, total WMH burden, DSWMH, and PVWMH. | | |
| --- | --- | --- |
| Variable | W | P.value |
| The number of affected anatomical regions | 0.908 | ＜0.001 |
| Total WMH burden | 0.847 | ＜0.001 |
| DSWMH | 0.819 | ＜0.001 |
| PVWMH | 0.816 | ＜0.001 |
| Abbreviations: WMH, white matter hyperintensities; PVWMH, Periventricular WMH; DWMH, Deep WMH; W, All W statistics represent Shapiro-Wilk normality test outputs. | | |
