# Supplementary material for: The association between body mass index and white matter hyperintensities: a cross-sectional analysis in a Tibetan population
Source: Front Neurol. 2026 Oct 22;17:1805682. doi: 10.3389/fneur.2026.1805682 (PMC13761218; doi:10.3389/fneur.2026.1805682)
Supplement: Supplementary file 5 [file Table_2.DOCX]

| Table S2 WMH anatomical distribution among 499 participants. | | |
| --- | --- | --- |
| WMH anatomical distribution | N | Percentage (%) |
| Periventricular | 405 | 81.2 |
| Frontal lobe | 412 | 82.6 |
| Parietal lobe | 267 | 53.5 |
| Temporal lobe | 63 | 12.6 |
| Occipital lobe | 77 | 15.4 |
| Centrum semiovale | 123 | 24.6 |
| Corona radiata | 24 | 4.8 |
| Internal capsule | 20 | 4.0 |
| External capsule | 38 | 7.6 |
| Brain stem | 17 | 3.4 |
| Significant WMH | 139 | 27.8 |
| Abbreviations: WMH, white matter hyperintensities; | | |
