# Supplementary material for: The association between body mass index and white matter hyperintensities: a cross-sectional analysis in a Tibetan population
Source: Front Neurol. 2026 Oct 22;17:1805682. doi: 10.3389/fneur.2026.1805682 (PMC13761218; doi:10.3389/fneur.2026.1805682)
Supplement: Supplementary file 6 [file Table_3.DOCX]

| Table S3 Characteristics of the 499 study participants stratified by WMH burden. | | | | |
| --- | --- | --- | --- | --- |
| Variables | Total | Non-significant WMH burden | Significant WMH burden | |
|  | (n = 499) | (n=360) | (N=139) | P |
| Age, year | 57.7 ± 11.7 | 54.7 ± 11.0 | 65.3 ± 9.7 | < 0.001 |
| Altitude, meters | 3746.2 ± 481.6 | 3759.0 ± 485.0 | 3713.1 ± 472.6 | 0.34 |
| HGB, g/L | 148.1 ± 24.2 | 148.0 ± 23.9 | 148.3 ± 25.2 | 0.909 |
| BMI, kg/m2 | 26.1 ± 4.0 | 26.4 ± 4.0 | 25.4 ± 3.7 | 0.012 |
| Sex, n (%) |  |  |  | 0.085 |
| Female | 221 (44.3) | 168 (46.7) | 53 (38.1) |  |
| Male | 278 (55.7) | 192 (53.3) | 86 (61.9) |  |
| Smoking status, n (%) | |  |  | 0.004 |
| Never | 307 (61.5) | 237 (65.8) | 70 (50.4) |  |
| Former | 105 (21.0) | 64 (17.8) | 41 (29.5) |  |
| Current smokers | 87 (17.4) | 59 (16.4) | 28 (20.1) |  |
| Alcohol status, n (%) | |  |  | < 0.001 |
| Never | 283 (56.7) | 219 (60.8) | 64 (46.0) |  |
| Former | 127 (25.5) | 74 (20.6) | 53 (38.1) |  |
| Current drinkers | 89 (17.8) | 67 (18.6) | 22 (15.8) |  |
| Hypertension, n (%) | |  |  | < 0.001 |
| No | 250 (50.1) | 211 (58.6) | 39 (28.1) |  |
| Yes | 249 (49.9) | 149 (41.4) | 100 (71.9) |  |
| Diabetes, n (%) |  |  |  | 0.784 |
| No | 431 (86.4) | 310 (86.1) | 121 (87.1) |  |
| Yes | 68 (13.6) | 50 (13.9) | 18 (12.9) |  |
| CAD, n (%) |  |  |  | 0.044 |
| No | 475 (95.2) | 347 (96.4) | 128 (92.1) |  |
| Yes | 24 ( 4.8) | 13 (3.6) | 11 (7.9) |  |
| Stroke, n (%) |  |  |  | < 0.001 |
| No | 448 (89.8) | 335 (93.1) | 113 (81.3) |  |
| Yes | 51 (10.2) | 25 (6.9) | 26 (18.7) |  |
| Antihypertensive therapy, n (%) | |  |  | < 0.001 |
| No | 340 (68.1) | 273 (75.8) | 67 (48.2) |  |
| Yes | 159 (31.9) | 87 (24.2) | 72 (51.8) |  |
| The data are shown as the mean ± standard deviation, median (interquartile range), or frequency (%), as appropriate. | | | | |
| Abbreviations: HGB, hemoglobin; CAD, coronary artery disease; WMH, white matter hyperintensities; BMI, body mass index; | | | | |
