# Supplementary material for: The association between body mass index and white matter hyperintensities: a cross-sectional analysis in a Tibetan population
Source: Front Neurol. 2026 Oct 22;17:1805682. doi: 10.3389/fneur.2026.1805682 (PMC13761218; doi:10.3389/fneur.2026.1805682)
Supplement: Supplementary file 7 [file Table_4.DOCX]

**Table S4. Product-of-Coefficients Mediation Analysis of BMI on Severe WMH (Fazekas 0–2 vs 3–6)**

| **Mediator** | **Path a (BMI→Med)** | **p_a** | **Path b (Med→WMH)** | **p_b** | **Indirect effect** | **95% CI** | **p** |
| --- | --- | --- | --- | --- | --- | --- | --- |
| Triglycerides | 0.0237 | 0.002 | −0.0405 | 0.838 | −0.0010 | −0.0107, 0.0078 | 0.838 |
| HDL cholesterol | −0.0074 | 0.011 | −0.1461 | 0.760 | 0.0011 | −0.0069, 0.0108 | 0.762 |
| Hypertension | 0.0405 | 0.194 | 0.7015 | 0.026 | 0.0284 | −0.0212, 0.0779 | 0.262 |

Note: Path a = coefficient for BMI → mediator; Path b = coefficient for mediator → severe WMH, adjusted for BMI and all covariates. Indirect effect = a × b. Models adjusted for age, sex, smoking, alcohol, CAD, stroke, diabetes, and antihypertensive therapy. Hypertension was modeled as binary (logistic path a); TG and HDL-C as continuous (OLS path a). Bootstrap CI based on 5,000 resamples.
