# Supplementary material for: The association between body mass index and white matter hyperintensities: a cross-sectional analysis in a Tibetan population
Source: Front Neurol. 2026 Oct 22;17:1805682. doi: 10.3389/fneur.2026.1805682 (PMC13761218; doi:10.3389/fneur.2026.1805682)
Supplement: Supplementary file 8 [file Table_5.DOCX]

**Table S5. Interaction and Stratified Analyses for BMI–WMH Association in Deep Brain Regions**

| **Analysis** | **Region** | **Term** | **OR** | **95% CI** | **p** |
| --- | --- | --- | --- | --- | --- |
| BMI × Altitude | Corona radiata | BMI × Altitude | 0.9999 | 0.9995–1.0003 | 0.556 |
| BMI × Altitude | Brainstem | BMI × Altitude | 0.9998 | 0.9994–1.0001 | 0.228 |
| HGB-stratified (Low) | Corona radiata | BMI | 0.735 | 0.600–0.900 | 0.003 |
| HGB-stratified (Low) | Brainstem | BMI | 0.739 | 0.597–0.915 | 0.005 |
| HGB-stratified (High) | Corona radiata | BMI | 0.783 | 0.645–0.952 | 0.014 |
| HGB-stratified (High) | Brainstem | BMI | 0.721 | 0.558–0.933 | 0.013 |

Note: BMI × Altitude interaction models adjusted for age, sex, smoking, alcohol, CAD, stroke, hypertension, diabetes, antihypertensive therapy, and HGB. HGB-stratified models used median split (147 g/L) with parsimonious covariates (age, sex, hypertension) due to low event counts. Low HGB: n = 249; High HGB: n = 250.
