# Supplementary material for: The association between body mass index and white matter hyperintensities: a cross-sectional analysis in a Tibetan population
Source: Front Neurol. 2026 Oct 22;17:1805682. doi: 10.3389/fneur.2026.1805682 (PMC13761218; doi:10.3389/fneur.2026.1805682)
Supplement: Supplementary file 9 [file Table_6.DOCX]

**Supplementary Table S6. Primary discharge diagnosis of study participants, stratified by body mass index category (n = 499).**

| **Admission diagnosis category** | **Normal weight (n=98)** | **Overweight (n=106)** | **Obese**  **(n=295)** | **P value** |
| --- | --- | --- | --- | --- |
| Total | 98 (100.0) | 106 (100.0) | 295 (100.0) |  |
| Cerebrovascular disease | 34 (34.7) | 32 (30.2) | 95 (32.2) | 0.794 |
| Cognitive impairment | 1 (1.0) | 2 (1.9) | 4 (1.4) | 0.872 |
| Epilepsy | 4 (4.1) | 2 (1.9) | 11 (3.7) | 0.659 |
| Parkinson's disease and parkinsonian syndromes | 8 (8.2) | 6 (5.7) | 16 (5.4) | 0.609 |
| Peripheral neuropathy and muscle disease | 3 (3.1) | 7 (6.6) | 19 (6.4) | 0.478 |
| Headache | 25 (25.5) | 17 (16.0) | 47 (15.9) | 0.097 |
| Dizziness | 1 (1.0) | 13 (12.3) | 24 (8.1) | 0.004 |
| Mood disorders and other conditions | 19 (19.4) | 23 (21.7) | 61 (20.7) | 0.929 |
| Other neurological diseases | 3 (3.1) | 4 (3.8) | 18 (6.1) | 0.468 |

Data are n (column %). Admission diagnoses were classified, according to each participant’s first discharge diagnosis, into nine groups. Body mass index (BMI) was classified using the WHO Western Pacific Region criteria: normal weight, < 23.0 kg/m²; overweight, 23.0–24.9 kg/m²; and obese, ≥ 25.0 kg/m². “Cerebrovascular disease” comprises ischaemic stroke or transient ischaemic attack, intracerebral haemorrhage, cervical or cerebral artery stenosis or atherosclerosis, and cerebral small vessel disease or vascular cognitive impairment. “Other neurological diseases” includes amyotrophic lateral sclerosis, peripheral facial palsy, herpes zoster, insomnia together with other neurological diagnoses. “Mood disorders and other conditions” comprises primary psychiatric (affective) diagnoses together with non-neurological conditions recorded as the first discharge diagnosis. P values were obtained from exact Fisher exact tests comparing each diagnostic category across the three BMI strata. Only dizziness differed significantly across BMI categories (P = 0.004), being considerably less frequent in normal-weight participants (1.0%) than in overweight (12.3%) or obese (8.1%) participants, whereas the proportion discharged with cerebrovascular disease was similar across the three groups (34.7%, 30.2% and 32.2%). Abbreviations: BMI, body mass index; TIA, transient ischaemic attack.
